# Outcomes and affecting factors for ICSI and microTESE treatments in nonobstructive azoospermia patients with different etiologies: A retrospective analysis

**DOI:** 10.3389/fendo.2022.1006208

**Published:** 2022-10-17

**Authors:** Songzhan Gao, Xianfeng Yang, Xiaoshuai Xiao, Shujun Yin, Yichun Guan, Jianhuai Chen, Yun Chen

**Affiliations:** ^1^ Department of Andrology, Jiangsu Province Hospital of Chinese Medicine, Affiliated Hospital of Nanjing University of Chinese Medicine, Nanjing, China; ^2^ Department of Andrology, The Third Affiliated Hospital of Zhengzhou University, Zhengzhou, China; ^3^ Department of Reproductive Medicine, The Third Affiliated Hospital of Zhengzhou University, Zhengzhou, China; ^4^ The First Affiliated Hospital of Henan University of Chinese Medicine, Zhengzhou, China

**Keywords:** nonobstructive azoospermia, microdissection testicular sperm extraction, intracytoplasmic sperm injection, sperm retrieval rate, pregnancy outcomes

## Abstract

**Introduction:**

Nonobstructive azoospermia (NOA) is a common and severe form of male infertility. Microdissection testicular sperm extraction (microTESE) combined with intracytoplasmic sperm injection (ICSI) is an optimal treatment for men with NOA. However, the outcomes and affecting factors of ICSI for NOA patients with different etiologies receiving microTESE treatment are still unclear.

**Methods:**

A total of 335 NOA patients undergoing microTESE from January 2017 to December 2021 were included in this retrospective analysis. The patients were divided into five groups (idiopathic, Klinefelter syndrome (KS), Y chromosome microdeletions (YCMDs), cryptorchidism and mumps orchitis) according to the etiologies. The clinical characteristics and outcomes of microTESE and ICSI were collected and comparisons were performed between clinical characteristics of patients who had successful sperm retrieval (SSR) and sperm retrieval failure (SRF). In addition, relationships between clinical characteristics and rates of SSR were explored by Kendall correlation analysis.

**Results:**

The overall SSR rate was 40.90%. SSR rate of the idiopathic group (31.22%) was the lowest and was much lower than that of other groups (KS: 48.65%, 28/58; YCMDs: 60.87%; cryptorchidism: 80.95%; mumps orchitis: 75.00%). The overall fertilization rate was 72.26%. No group differences were found among five groups (idiopathic: 73.91%; KS: 71.43%; YCMDs: 64.29%; cryptorchidism: 70.59%; mumps orchitis: 77.78%). The overall clinical pregnancy rate was 66.67%. No group differences were found among five groups (idiopathic: 68.63%; KS: 65.00%; YCMDs: 44.44%; cryptorchidism: 66.67%; mumps orchitis: 85.71%). The overall live birth rate was 66.67%. No group differences were found among five groups (idiopathic: 71.43%; KS: 53.85%; YCMDs: 50.00%; cryptorchidism: 75.00%; mumps orchitis: 66.67%). For SSR patients, the average age was significantly lower in the idiopathic group, while the average testicular volume was significantly greater in the cryptorchidism and mumps orchitis groups. However, no significant differences were found in the level of follicle stimulating hormone (FSH), luteinizing hormone (LH) and testosterone (T) between patients who had SSR and SRF. In addition, negative relationships were found between age and rates of SSR in idiopathic NOA patients while positive relationships were found between testis volume and rates of SSR in patients with cryptorchidism and mumps orchitis.

**Conclusion:**

Patients with idiopathic NOA had lowest SSR. In addition, the age in idiopathic NOA patients was a predictor for SSR while testicular volume in NOA patients with cryptorchidism and mumps orchitis was a predictor for SSR. However, the relationships between clinical characteristics and clinical outcomes in NOA patients were preliminary, and further validation needed to be carried out in a larger sample to increase statistical capacity before a definitive conclusion could be drawn.

## Introduction

Infertility is a common condition affecting approximately 15% of couples who try to conceive (1, 2). Male factors are responsible for about 50% of these cases (3). Azoospermia is a major cause and is the most severe phenotype of male infertility, which occurs in approximately 10%-20% of infertile men seeking medical care for infertility (4, 5). Nonobstructive azoospermia (NOA) is a common and severe phenotypic manifestation of male infertility patients with azoospermia, which accounts for about 1% of the male population and approximately 10% of infertile couples (6, 7). NOA is characterized by a complete absence of spermatozoa in semen without any obstructive factors and is considered to be caused by fully or partly spermatogenic dysfunction, which ranges from hypospermatogenesis (HS) and maturation arrest (MA) to Sertoli cell-only syndrome (SCOS) (8, 9). Possible aetiologies of NOA include genetic factors including Klinefelter syndrome (KS) and Y chromosome microdeletions (YCMDs), congenital abnormalities including cryptorchidism, idiopathic factors and acquired causes, such as postinfectious including mumps orchitis (6).

The clinical evaluation and management of patients with NOA has been a challenge for andrologists and reproductive medicine specialists (10). The management of NOA patients have been revolutionized with the introduction of technique of intracytoplasmic sperm injection (ICSI) (11). For NOA patients, the primary goal is to obtain viable spermatozoa that can be used for artificial reproductive technologies (12, 13). Different methods have been developed for obtaining viable spermatozoa in NOA patients, which include conventional testicular sperm extraction (cTESE) and microdissection testicular sperm extraction (microTESE) (14, 15). The combination of TESE and ICSI has become the first-line treatment for patients with azoospermia (16). One or multiple biopsies are taken blindly via small incisions of the testis during cTESE while the tunica albuginea is widely opened and the testicular tissue is examined at ×20-25 magnification, which allows for retrieval of more number of sperm cells and can improve the successful sperm retrieval (SSR) rate in patients with NOA in microTESE (14). Therefore, the method of microTESE appears to be more effective than cTESE for the retrieval of spermatozoa in NOA patients.

However, the outcomes of microTESE in NOA patients are difficult to predict based on the existing variables and the clinical value of predictors found in previous studies is limited (17, 18). So far, there are some controversies over the outcomes (including SSR and clinical pregnancy) and affecting factors for ICSI and microTESE treatments in NOA patients with different etiologies. The present study aimed to compare the outcomes of microTESE and ICSI treatments for NOA patients with different etiologies (idiopathic, KS, YCMDs, cryptorchidism and mumps orchitis) retrospectively. In addition, determinant factors (clinical characteristics) for microTESE outcomes were compared between patients who had SSR and sperm retrieval failure (SRF).

## Materials and methods

### Patients

In this study, a total of 335 NOA patients who underwent microTESE in an attempt to find sperms for ICSI were included from January 2017 to December 2021 in the Department of Andrology, The Third Affiliated Hospital of Zhengzhou University, Zhengzhou, China. The study protocol was approved by the Ethics Committee of The Third Affiliated Hospital of Zhengzhou University. In addition, all patients provided written informed consent.

The inclusion criteria for azoospermia patients were as follows: (1) absence of ejaculated sperm observed in at least three semen samples after centrifuge and screening using an inverted microscope according to the World Health Organization fifth edition guidelines; (2) normal ejaculate volume and pH; (3) no sign of obstruction of the seminal tract evaluated by physical examination, scrotal and transrectal ultrasound; (4) had available clinical data, including medical history, physical examination, assessments of hormones, scrotal ultrasound, genetic testing.

The exclusion criteria were as follows: (1) serious physical disease; (2) serious mental diseases; (3) serious female infertility factors, including anovulation, tubal factors, polycystic ovary syndrome, hormonal and immunological infertility, ovarian failure and endometriosis.

The patients were divided into five groups (idiopathic, KS, YCMDs, cryptorchidism and mumps orchitis) according to the etiologies. Patients were diagnosed with idiopathic NOA on the basis of comprehensive andrological testing, including examination of medical history, physical examination, semen analysis, scrotal ultrasound, sex hormone assessments, chromosomal karyotyping and Y chromosome microdeletion analyses, whole-exome sequencing analysis. However, no clear pathogenic factors associated with azoospermia were found. All the 58 NOA patients with KS were non-mosaic Klinefelter man while all the 23 NOA patients with YCMDs had partial deletions of AZFc region on the Y chromosome. In 21 NOA patients with cryptorchidism, more than 90% (n=19) of the undescended testes were located in the inguinal region while the intra-abdominal location accounted for less than 10% (n=2) of the cases. In addition, all testes were brought down into the scrotum by orchiopexy prior to the SSR and at the age of (18.33 ± 3.86) years. The demographic, clinical and laboratory data including age, hormonal profile for follicle stimulating hormone (FSH), luteinizing hormone (LH) and testosterone (T), bilateral testicular volume of all patients were collected (**Table 1**). In addition, outcomes of microTESE including rates of SSR and outcomes of ICSI including rates of fertilization, clinical pregnancy, live birth were acquired.

**Table 1:** Clinical characteristics of NOA patients with different etiologies.

Characteristics	Whole cohort(n=335)	Idiopathic(n=221)	KS(n=58)	YCMDs(n=23)	Cryptorchidism(n=21)	Mumps orchitis(n=12)
Age (years)	31.53 ± 4.11	32.09 ± 3.52	31.17 ± 4.86	30.78 ± 6.22	28.48 ± 4.06	29.58 ± 2.43
FSH (IU/L)	18.90 ± 15.08	14.03 ± 12.17	38.15 ± 14.86	15.65 ± 8.05	19.26 ± 5.59	21.19 ± 12.20
LH (IU/L)	10.76 ± 8.31	7.49 ± 5.21	23.04 ± 9.15	8.46 ± 4.00	12.72 ± 4.03	12.51 ± 4.99
T (ng/mL)	9.51 ± 6.04	10.11 ± 6.46	5.35 ± 4.22	12.38 ± 3.98	10.59 ± 1.06	11.32 ± 5.04
LTV (mL)	6.21 ± 3.38	7.37 ± 3.19	2.10 ± 0.72	7.52 ± 2.11	4.71 ± 1.38	4.75 ± 1.66
RTV (mL)	6.23 ± 3.39	7.34 ± 3.20	2.09 ± 0.78	7.78 ± 2.28	5.24 ± 1.30	4.50 ± 1.73

NOA, nonobstructive azoospermia; FSH, follicle stimulating hormone; LH, luteinizing hormone; T, testosterone; LTV, left testicular volume; RTV, right testicular volume; KS, klinefelter syndrome; YCMDs, Y chromosome microdeletions.

### Surgical procedure of microTESE

MicroTESE was performed by the same experienced andrologist under general anesthesia, as described in previous study (19). The procedure started in the larger testicle after scrotal disinfection in the supine position. The testicular parenchyma was opened by a mid-line scrotal incision without affecting of blood supply. In addition, this procedure should be performed at minimizing tissue stretching and preserving the caliber of the underlying seminiferous tubules. Then the testicular parenchyma was directly examined to locate and collect tubules that appeared clearly dilated compared to the surroundings (the wider seminiferous tubules and larger and whiter tubules) at ×20-25 magnification by an operating microscope with higher chance of harboring spermatozoa. Dilated tubules (2-10 mg) were obtained and placed in petri dishes containing human tubular fluid, taking into account how to cause the least vascular damage. The sperm were given and assessed by an experienced embryologists for the immediate identification of spermatozoa. The procedure was terminated when suitable sperm were successfully retrieved or when further dissection might likely damage the blood supply of testicle. The similar procedure was performed on the contralateral testis if no spermatozoa were retrieved. At the same time, a piece of testicular tissues was obtained and fixed in Bouin’s solution and sent for histopathological examination.

### Sperm processing

Testicular fragments were washed to remove the blood and were placed in the sterile tissue culture dishes with sperm washing medium. Then the washed testicular fragments were finely minced using micro scissors. The resulting samples were changed into a homogeneous pulverized suspension. The small aliquots of suspension were then directly examined for the presence of spermatozoa at ×200 magnification by an inverted microscope. If recoverable sperm was not found on the day of surgery, the search continued for the remaining cell suspensions on the next morning. If fresh oocytes were obtained on the same or the next day of sperm retrieval, fresh sperm were used for the ICSI. If not, sperm was routinely cryopreserved with sperm-freezing solution. The samples were mixed 1:1 with equal volume of sperm freezing medium and equilibrated for 10 minutes at room temperature. And then these samples were placed above the liquid nitrogen for half an hour. Finally, the resulting samples were immersed in liquid nitrogen and transferred to the sperm storage bank. When thawing, the sample was transferred to room temperature for 5 minutes. The sperm were collected from the cryoprotectant by washing in culture medium and centrifugation at 2,000 rpm for minutes. The resulting samples were resuspended in culture medium for later use. The details about sperm processing could be found in previous study (20).

### Ovarian stimulation and oocyte retrieval and ICSI

The ovarian stimulation protocol combined the use of gonadotrophin-releasing hormone (GnRH) analogs, FSH, and human chorionic gonadotrophin. Oocyte retrieval was carried out using vaginal ultrasound-guided puncture at 36-38 hours after HCG administration. The obtained oocytes were washed with buffer and inseminated liquid and were cultured for 2 hours at a 37°C incubator with 6%CO_2_, 5%O_2_ and 95% humidity. The surrounding cumulus cells were removed at 2 hours after retrieval by pipetting and exposure to hyaluronidase and then ICSI was performed after 1 hour. The fertilized eggs were cultured at 37°C, 6%CO_2_, 5%O_2_ and 95% humidity for 3 to 5 days, and the well-developed embryos or blastocysts were selected for transfer. Hormone replacement therapy or natural cycles were used for the endometrial preparation. Embryo resuscitation was performed on the 6th day after endometrial transformation or the 5th day after ovulation. Embryo transfer was conducted under the guidance of ultrasound, and 1 to 2 embryos were transferred each time to reduce the risk of a multiple pregnancy. The details about ovarian stimulation and oocyte retrieval and ICSI could be found in previous studies (21, 22).

### Definitions of ICSI outcomes

Clinical outcomes included the fertilization, clinical pregnancy and live birth rates. Fertilization was identified by the presence of two pronuclei (2pn) and two polar bodies after the intracytoplasmic injection of motile spermatozoa. Pregnancy was defined as a spontaneous rise in the serum HCG level at least 10 days after embryo transfer. Clinical pregnancy was determined by the presence of an intrauterine gestational sac by ultrasound examination at the 5th week after embryo transfer.

### Statistical analysis

In this study, the statistical analysis was performed using the Statistical Package for the Social Sciences version 23.0 (SPSS Inc, Chicago, IL, United States). The continuous variables were expressed as means ± standard deviation (SD) while categorical variables were expressed as proportions (%). The distribution of data was evaluated by Kolmogorov Smirnov test while the homogeneity of variance was evaluated using Levene test. Group differences of demographic and clinical data were compared by on way analysis of variance (ANOVA) with *post hoc* contrasts by least significant difference (LSD) test for continuous variables and Chi-square test for categorical variables. In addition, relationships between clinical characteristics and rates of SSR were explored by Kendall correlation analysis. *P*<0.05 was considered statistically significant.

## Results

### Comparison outcomes of microTESE among NOA patients with different etiologies

The overall SSR rate was 40.90% (137/335). SSR rate of the idiopathic group (31.22%, 69/221) was the lowest and was much lower than that of other groups (KS: 48.65%, 28/58; YCMDs: 60.87%, 14/23; cryptorchidism: 80.95%, 17/21; mumps orchitis: 75.00%, 9/12; χ^2 ^= 33.37; P<0.01) (**Tables 2**, **3**).

**Table 2:** Outcomes of NOA patients with different etiologies who underwent microTESE for ICSI.

Variables (n/%)	Whole cohort(n=335)	Idiopathic(n=221)	KS(n=58)	YCMDs(n=23)	Cryptorchidism(n=21)	Mumps orchitis(n=12)
Successful sperm retrieval	137 (40.90%)	69 (31.22%)^*^	28 (48.65%)	14 (60.87%)	17 (80.95%)	9 (75.00%)
Fertilization	99 (72.26%)	51 (73.91%)	20 (71.43%)	9 (64.29%)	12 (70.59%)	7 (77.78%)
Clinical pregnancy	66 (66.67%)	35 (68.63%)	13 (65.00%)	4 (44.44%)	8 (66.67%)	6 (85.71%)
Live birth	44 (66.67%)	25 (71.43%)	7 (53.85%)	2 (50.00%)	6 (75.00%)	4 (66.67%)

NOA, nonobstructive azoospermia; microTESE, microdissection testicular sperm extraction; ICSI, intracytoplasmic sperm injection; KS, klinefelter syndrome; YCMDs, Y chromosome microdeletions. The fertilization rate was referred to the number of patients with successful sperm retrieval. The clinical pregnancy was relative to the fertilization rate while the live birth was relative to the clinical pregnancy rate. ^*^ indicated significant differences between gropus.

**Table 3:** Predictors of successful sperm retrieval for NOA patients with different etiologies.

SSR predictors	Whole cohort(40.90%)SSR(137)/SRF(198)	Idiopathic(31.22%)SSR(69)/SRF(152)	KS(48.65%)SSR(28)/SRF(30)	YCMDs(60.87%)SSR(14)/SRF(9)	Cryptorchidism(80.95%)SSR(17)/SRF(4)	Mumps orchitis(75.00%)SSR(9)/SRF(3)
Age (years)	30.96 ± 4.27*/31.92 ± 3.96*	31.35 ± 3.50*/32.43 ± 3.49*	31.64 ± 4.86/30.73 ± 4.91	32.14 ± 6.33/28.67 ± 5.72	28.12 ± 3.79/30.00 ± 5.42	29.33 ± 2.74/30.33 ± 1.155
FSH (IU/L)	20.36 ± 14.74/17.88 ± 15.26	14.57 ± 12.86/13.78 ± 11.88	36.66 ± 14.52/39.54 ± 15.28	16.70 ± 7.83/14.03 ± 8.57	19.66 ± 6.05/17.55 ± 2.93	21.09 ± 12.64/21.50 ± 13.39
LH (IU/L)	11.79 ± 8.31/10.04 ± 8.25	7.55 ± 4.88/7.46 ± 5.37	23.18 ± 8.52/22.92 ± 9.85	8.54 ± 4.08/8.35 ± 4.12	12.77 ± 4.44/12.51 ± 1.71	12.12 ± 5.35/13.67 ± 4.42
T (ng/dL)	9.71 ± 5.87/9.37 ± 6.17	10.42 ± 6.67/9.96 ± 6.38	5.32 ± 4.21/5.37 ± 4.31	12.81 ± 4.29/11.71 ± 3.58	10.52 ± 1.11/10.85 ± 0.85	11.61 ± 4.58/10.43 ± 7.39
LTV (mL)	6.14 ± 3.22/6.26 ± 3.50	7.78 ± 2.91/7.18 ± 3.30	2.035714 ± 0.74/2.17 ± 0.70	7.93 ± 2.16/6.89 ± 1.96	5.18 ± 1.01*/2.75 ± 0.96*	5.33 ± 1.41*/3.00 ± 1.00*
RTV (mL)	6.20 ± 3.22/6.247 ± 3.51	7.72 ± 2.97/7.17 ± 3.29	2.14 ± 0.80/2.03 ± 0.76	8.21 ± 2.29/7.11 ± 2.20	5.65 ± 1.06*/3.50 ± 0.58*	5.11 ± 1.45*/2.67 ± 1.15*

NOA, nonobstructive azoospermia; FSH, follicle stimulating hormone; LH, luteinizing hormone; T, testosterone; LTV, left testicular volume; RTV, right testicular volume; KS, klinefelter syndrome; YCMDs, Y chromosome microdeletions; SSR, successful sperm retrieval; SRF, sperm retrieval failure. ^*^ indicated significant differences of clinical characteristics between patients who experienced SSR and SFR.

For SSR patients, the average age (t=-2.13; P=0.034) was significantly lower in the idiopathic group, while the average testicular volume was significantly greater in the cryptorchidism (left: t=4.34, P=0.00035; right: t=3.88, P=0.001) and mumps orchitis (left: t=2.61, P=0.026; right: t=2.62, P=0.026) groups. However, no significant differences were found in the level of follicle stimulating hormone (FSH), luteinizing hormone (LH) and testosterone (T) between patients who had SSR and SRF (**Table 3**).

### Comparison outcomes of ICSI among NOA patients with different etiologies

In this study, the fertilization rate was referred to the number of patients with successful sperm retrieval. The clinical pregnancy was relative to the fertilization rate while the live birth was relative to the clinical pregnancy rate.

The overall fertilization rate was 72.26% (99/137). No group differences were found among five groups (idiopathic: 73.91%, 51/69; KS: 71.43%, 20/28; YCMDs: 64.29%, 9/14; cryptorchidism: 70.59%, 12/17; mumps orchitis: 77.78%, 7/9; χ^2 ^= 0.71; P=0.95) (**Table 2**).

The overall clinical pregnancy rate was 66.67% (66/99). No group differences were found among five groups (idiopathic: 68.63%, 35/51; KS: 65.00%, 13/20; YCMDs: 44.44%, 4/9; cryptorchidism: 66.67%, 8/12; mumps orchitis: 85.71%, 6/7; χ^2 ^= 3.26; P=0.52) (**Table 2**).

The overall live birth rate was 66.67% (44/66). No group differences were found among five groups (idiopathic: 71.43%, 25/35; KS: 53.85%, 7/13; YCMDs: 50.00%, 2/4; cryptorchidism: 75.00%, 6/8; mumps orchitis: 66.67%, 4/6; χ^2 ^= 2.07; P=0.75) (**Table 2**).

### Relationships between age, testis volume and SSR in NOA patients receiving microTESE.

Negative relationships were found between age and rates of SSR in idiopathic NOA patients (τ=-0.12; P=0.028) while positive relationships were found between testis volume and rates of SSR in patients with cryptorchidism (τ=0.63; P=0.0028; left: τ=0.63; P=0.0028) and mumps orchitis (left: τ=0.63; P=0.025; left: τ=0.62; P=0.028).

## Discussion

In the present study, we aimed to explore the outcomes and affecting factors for ICSI and microTESE treatments in nonobstructive azoospermia patients with different etiologies retrospectively. The results showed that the overall SSR rate was 40.90% for all NOA patients. The idiopathic NOA group had the lowest SSR rate in the five groups. The overall fertilization, clinical pregnancy and live birth rate was 72.26%, 66.67% and 66.67% for all NOA patients respectively. In addition, for SSR patients, idiopathic NOA patients were younger, and NOA patients with cryptorchidism and mumps orchitis had larger testicle than those who had SRF. Moreover, negative relationships were found between age and rates of SSR in idiopathic NOA patients while positive relationships were found between testis volume and rates of SSR in patients with cryptorchidism and mumps orchitis. These findings suggested that idiopathic factors might be predictive of lower SSR rate while age and testicular volume might be predictive of higher SSR rate. However, to predict preoperatively SSR from NOA patients remained challenging.

It has established that TESE is the recommended method for sperm retrieval in NOA patients (23, 24). A number of previous studies have compared the outcomes and affecting factors between NOA patients who have underwent cTESE and microTESE treatments followed by ICSI (14, 25, 26). The results of a meta-analysis demonstrated that SSR rate was higher in NOA patients who received the treatment of microTESE when compared with those who treated with cTESE (14). The SSR rate of cTESE ranged from 35% to 56% in NOA patients and these patients was 1.5 times more likely to obtain viable spermatozoa with the treatment of microTESE when compared with those undergone cTESE, which suggested that microTESE was better than cTESE for SSR (6). The SSR rate was also reported to range from 16.7% to 45% in the cTESE group and 42.9% to 63% in the microTESE group, which were positively related to the level of FSH and testicular volume (27). Another meta-analysis showed that SSR rate was up to 50% in all NOA patients who received the treatment of cTESE/mTESE and no differences of SSR rate were found between these two groups (11).

In the present study, the overall SSR rate of all NOA patients was 40.90%. In addition, the sperm retrieval was least successful in the idiopathic group (31.22%), followed by KS (48.65%), YCMDs (60.87%) and mumps orchitis (75.00%), and was most successful in the cryptorchidism group (80.95%). In addition, idiopathic NOA patients with SSR were younger than those who had SRF. A retrospective review suggested that no individual clinical characteristic was found to accurately predict the sperm retrieval of microTESE in NOA patients (28). In addition, age was found have no impacts on the sperm retrieval rates of microTESE in a retrospective study (29). Conversely, another previous study had demonstrated that the outcomes of TESE procedure performed at early age were better than those with TESE performed in older (30). In consistent with this view, our findings showed that age was a favorable factor for SSR in idiopathic NOA patients. Besides age, other factors including testicular volume had been advocated as possible prognostic values for SSR in NOA patients (31). In particular, the large volume of testicle (more than 12 ml) might lead to a SSR rate greater than 60% with an accuracy of 86% (11). Our data also showed that the SSR rate was influenced by the testicular volume in NOA patients, especially for those with cryptorchidism and mumps orchitis. Therefore, the age and testis volume might be the significant predictive factors of SSR for NOA patients. In addition, the level of FSH and testosterone were considered to be the most influential predictive factors for sperm retrieval of microTESE in NOA patients (32). However, in this study, the level of hormone including FSH, LH and T were not related to the sperm retrieval rate of microTESE, which was consisted with previous study (33).

Previous studies on predictors of microTESE outcomes had primarily focused on SSR as the sole endpoint for success. In this study, the outcomes for ICSI microTESE treatments were also acquired in nonobstructive azoospermia patients with different etiologies. The overall fertilization, clinical pregnancy and live birth rate was 72.26%, 66.67% and 66.67% for all NOA patients respectively and no differences were found among groups. In previous study, the fertilization, implantation and clinical pregnancy rates were 54.2%, 5% and 23.1% in NOA patients (34). The clinical features and the level of hormone had no significant effects on the outcome of ICSI, including fertilization, implantation and pregnancy rate (34). Moreover, no differences were found between the outcome of ICSI with fresh and frozen-thawed spermatozoa in NOA patients (35). In addition, NOA patients with orchitis had the highest rates of fertilization, clinical pregnancy and live birth while patients with YCMDs had the lowest rates of fertilization and clinical pregnancy (36). The type of azoospermia was also an important predictor of successful clinical pregnancy for patients with azoospermia and patients with acquired NOA had the higher probability of successful clinical pregnancy (37). By contrast, NOA patients with congenital and idiopathic factors had lower likelihoods of achieving clinical pregnancy (38).

The reported SSR rates for NOA patients with YCMDs and KS were higher when compared to those in previous studies. The deletion of a specific region in Y chromosome could help predict the SSR rate. More than half of patients with c (AZFc) microdeletions had SSR, however, sperm was not found in men with patients with a and b (AZFa and AZFb) microdeletions (39). Since all patients with YCMDs had partial deletions of AZFc region on the Y chromosome, the reported SSR rate for patients withYCMDs in this study might be higher when compared to that of previous study. In addition, the SSR was about 30%-50% in NOA patients with KS (40). The age and mosaic KS might be favorable factors for SSR in KS patients receiving microTESE (41, 42). In this study, the average age of KS patients was younger and all patients were non-mosaic KS man, which were two favorable factors for SSR of KS patients. All these favorable factors might lead to higher SSR for patients with YCMDs and KS in this study.

In this study, the overall clinical pregnancy rate was 66.67% (idiopathic: 68.63%; KS: 65.00%; YCMDs: 44.44%; cryptorchidism: 66.67%; mumps orchitis: 85.71%) while the overall live birth rate was 66.67% (idiopathic: 71.43%; KS: 53.85%; YCMDs: 50.00%; cryptorchidism: 75.00%; mumps orchitis: 66.67%). In the previous study, the rates of clinical pregnancy and live birth were 46.9% and 40.6% in idiopathic NOA patients (n=319), 54.4% and 50.4% in NOA patients with KS (n=125), 20.3% and 18.8% in patients with YCMDs (n=91, 11.83%), 53.9% and 46.2% in patients with cryptorchidism (n=52), 78.3% and 74.0% in patients with mumps and bilateral orchitis (n=23) (36). In another study, the rates of clinical pregnancy were 55.84% and 50.97% in NOA patients receiving resh and frozen microTESE (43). In this study, the overall clinical pregnancy rate was 66.67% (idiopathic: 68.63%; KS: 65.00%; YCMDs: 44.44%; cryptorchidism: 66.67%; mumps orchitis: 85.71%) while the overall live birth rate was 66.67% (idiopathic: 71.43%; KS: 53.85%; YCMDs: 50.00%; cryptorchidism: 75.00%; mumps orchitis: 66.67%). Previous studies had demonstrated that NOA patients with AZFc microdeletions had the lowest clinical pregnancy rate and the lowest live birth rate when compared to patients with other etiologies, including idiopathic, KS, cryptorchidism and mumps orchitis (36, 44). In this study, the percentage of patients with AZFc microdeletions was 6.87%, which was lower than that of previous study (11.83%). Considering the low percentage of patients with AZFc microdeletions in the whole sample, the reported clinical pregnancy and live birth rate might be higher when compared with patients with high percentage of AZFc microdeletions in previous study. In addition, all NOA patients received resh microTESE in this study, however, both resh and frozen microTESE were applied in previous study. These two favorable factors might lead to the high clinical pregnancy and live birth rate in this study.

However, there were several limitations in this study. Firstly, the quality of demographic and clinical data might be affected by the retrospective nature of the study. Secondly, the relatively small sample size might reduce the statistical power to detect differences between groups. Finally, the limited clinical data might restrict the statistical power for detecting predictors for the outcomes of ICSI and microTESE treatments in NOA patients with different etiologies. Therefore, prospective studies with larger sample size and more clinical measures should be performed to explore the predictive factors for SSR of microTESE and clinical outcomes of ICSI in different NOA patients.

## Conclusion

Our findings suggested that the etiology was predictive of the SSR in NOA patients. Among all etiologies, idiopathic NOA patients had lowest SSR. Moreover, the age and testis volume were the significant predictive factors for SSR in idiopathic and acquired NOA patients respectively. These results emphasized the role of microTESE as a standard surgical method for retrieving spermatozoa in NOA patients and provided clinicians with strongly relevant guidance to inform clinical practice.

## Data availability statement

The raw data supporting the conclusions of this article will be made available by the authors, without undue reservation.

## Ethics statement

The studies involving human participants were reviewed and approved by the Ethics Committee of The Third Affiliated Hospital of Zhengzhou University. The patients/participants provided their written informed consent to participate in this study.

## Author contributions

SG, JC, YC, XY and YG designed the experiments. SG, XX, SY, XY and YG contributed to clinical data collection and assessment. SG, JC, YC and XY analyzed the results. SG and JC wrote the manuscript. All authors approved the final manuscript. All authors contributed to the article and approved the submitted version.

## Funding

The work was supported by the grants of: the 2021 Henan Medical Science and Technology Research Plan Joint Co-construction Project (No. LHGJ20210433).

## Conflict of interest

The authors declare that the research was conducted in the absence of any commercial or financial relationships that could be construed as a potential conflict of interest.

## Publisher’s note

All claims expressed in this article are solely those of the authors and do not necessarily represent those of their affiliated organizations, or those of the publisher, the editors and the reviewers. Any product that may be evaluated in this article, or claim that may be made by its manufacturer, is not guaranteed or endorsed by the publisher.
